# Dietary intake and risk assessment of nitrosamine in processed meat products among medical staff during their night shift

**DOI:** 10.1038/s41598-024-84059-y

**Published:** 2025-01-14

**Authors:** Dalia Ibrahim Tayel, Noorhan K. Farrag, Samar Mohamed Aborhyem

**Affiliations:** 1 Nutrition Department, High Institute of Public Health, Alexandria University, 165 EL-Horreya Avenue, EL-Hadarah, Alexandria, 21561 Egypt; 2 Food Analysis, Nutrition Department, High Institute of Public Health, Alexandria University, Alexandria, Egypt

**Keywords:** Dietary intake, Risk assessment, Margin of exposure, Nitrosamine, Medical staff, Night shift, Environmental sciences, Natural hazards, Health occupations

## Abstract

The study aims to evaluate the levels of nitrosamine, a known carcinogenic compound, in processed meat products and to assess its dietary intake and margin of exposure among medical staff, including physicians, pharmacists, and nurses working night shifts at Alexandria University Hospitals. Additionally, the study seeks to evaluate the participants’ knowledge of dietary sources and regulatory limits of carcinogens. A cross-sectional study was conducted with 420 participants. Results showed that hotdogs contained the highest nitrosamine levels (159.24 ± 87.99 µg/g) with a consumption pattern of 0.02126 µg/kg BW/day. In contrast, sausages and burgers had the lowest nitrosamine content (2.36 µg/g and 2.37 µg/g, respectively). Luncheon meat also exhibited low nitrosamine levels and consumption patterns, at 18.53 µg/g and 0.00141 µg/kg BW/day, respectively. The Margin of Exposure (MOE) was calculated to assess risk, with a Benchmark Dose Lower Confidence Limit (BMDL)10 value of 29 µg/kg BW/day as the reference point. MOE values ≥ 17,000, derived for nitrosamine exposure in this study, indicate a low level of concern for carcinogenic risk. A statistically significant difference in dietary nitrosamine intake was observed across different professions among the medical staff (*p* < 0.001).

**Supplementary Information:**

The online version contains supplementary material available at 10.1038/s41598-024-84059-y.

## Introduction

N-nitrosamines are chemical compounds formed when nitrites and nitrates react with secondary amines, especially in acidic environments like the human stomach, a reaction more likely under acidic conditions and high temperatures such as those during cooking. Nitrites and nitrates, commonly used as preservatives and color fixatives in processed meats. Many N-nitrosamines are potent carcinogens, capable of causing DNA mutations that lead to cancer, with dietary intake from processed meats being a significant exposure source. Regulatory bodies like the European Food Safety Authority (EFSA) and the U.S. Food and Drug Administration (FDA) have established guidelines and limits on nitrites and nitrates in food to minimize nitrosamine formation. Many countries, including the European Union, have set legal limits on the use of these compounds [Commission Regulation (EU) No. 601/2014]. Comprehensive risk assessments are conducted to evaluate the health risks of nitrosamine exposure, considering nitrite and nitrate levels, conditions promoting nitrosamine formation, and human exposure levels. Nitrates and nitrites are commonly used in cured meat products to preserve them and enhance their flavor. However, nitrites have been linked to the formation of cancer-causing N-nitroso-compounds. In the first half of the 20th century, nitrites replaced nitrates due to their quicker curing time, larger manufacturing capacity, and improved chemistry understanding. Nitrates are now only used in a few special products such as dry-cured hams and dry sausage due to their lengthy curing processes^1^.

Nitrites in meat are quickly reduced and converted into various substances such as nitrous acid, nitric oxide (NO), and nitrates^2^. These nitrates are primarily present in a dissociated form at a pH of 5.6 to 5.8 in meat. At this pH, they are transformed into several unstable intermediate chemicals that are difficult to identify. These nitrite-derived chemicals have oxidizing, reducing, and nitrosylating properties^2^. The reduction of nitrites in meat products may be due to the activity of meat-specific endogenous reducing agents such as cysteine (a sulfur-containing amino acid) or ascorbate additions^3^.

Ascorbate has been found to interact with the resulting NO, which can then interact with other components of meat. It is widely accepted that the majority of meat-curing reactions require the synthesis of NO from nitrites as the first step^3^. However, both short-term and long-term effects on human health are caused by nitrates and nitrites. Although nitrates are generally considered safe, their metabolic byproducts, including nitrites, NO, and N-nitroso compounds (NOCs), have raised concerns about potential negative health effects. Nitrites are about ten times more acutely toxic than nitrates. The lethal oral dosages for humans are 80–800 mg nitrates kg-1 bw and 33–250 mg nitrites kg-1 bw^4^. In Germany, in the early 20th century, excessive amounts of nitrites added to meat products resulted in fatalities due to poisoning^3^. Methemo-globinemia, which is characterized by cyanosis and is caused by high nitrite exposure, is the primary adverse effect associated with acute nitrite toxicity. Nitrites prevent oxygen from attaching to hemoglobin, making infants under three months of age more susceptible to this condition than older children and adults^5^.

The risk of nitrosamines (NA) in humans is evaluated using the margin of exposure approach (MOE)^6^. To determine the significance of the exposure, the dose in experimental animals that results in a specific incidence of tumor growth is compared with the predicted dietary exposure to genotoxic chemicals. As the margins of exposure (MOE) between the impact dosage level and the actual exposure level rise, the level of worry diminishes. A widely used toxicological reference point is the Benchmark Dose Lower Confidence Limit (BMDL), which indicates the exposure level at which an increase in the incidences of the effect is smaller than the specified Benchmark Response with a confidence level of 95% (at 10% in the case of animal experiments)^7^. The BMDL10 may be derived using dose-response data from several long-term carcinogenicity studies rather than a single complete study. If the BMDL10 is 10,000 times higher than the exposure (i.e., MOE of 10,000) for genotoxic chemicals, then there is minimal need for concern^8^.

Night labour has been identified as one of the most pervasive occupational characteristics in today’s culture^9^, and has been connected to several chronic illnesses including; cancer, metabolic disorders, and cardiovascular disease^10^. For instance, during night shifts, shift workers are more likely to break their usual eating routines^11^. Night shift workers are mainly dependent on fast food and snacks during their work hours because of the availability of this type of food more than other healthy foods. These food items have many concerns about their chemical composition due to the risk of containing nitrosamine which has been reported to have a negative health impact. This study aims to quantify nitrosamine levels in the most commonly consumed processed meat products from various brands available in hypermarkets. Additionally, it seeks to evaluate the dietary intake of nitrosamines among medical staff working night shifts and assess their awareness of carcinogens present in fast food meals.

## Materials and methods

### Part I

#### The sample size for the target population (physicians, pharmacists, and nurses)

Epi-Info version 7 was used for calculating the sample size. Based on the assumption that 50% of medical staff had carcinogenic compounds in their diet and a confidence limit of 5%, the minimal sample size at a 95% confidence level was calculated to be 384, which was rounded to 420.

#### Type of samples and methods of selection

The target population for this study included 420 participants comprising doctors, nurses, and pharmacists from various healthcare settings in Alexandria Governorate, Egypt. Participants were selected randomly based on their professional roles, frequent consumption of processed meats, and willingness to participate in the study. The age range was limited to individuals up to 39 years old to focus on the dietary habits of a younger demographic, as this group is more likely to consume fast food and processed meats frequently. Targeting this age group allows for a more accurate assessment of the impact of processed meat consumption on dietary intake of N-nitrosamines. Voluntary consent to participate in dietary surveys was required to ensure that participants were willing and able to provide accurate and detailed information about their eating habits, enhancing the reliability and validity of the data collected.

The predesigned self-reported questionnaire, administered between August 2021 and July 2022, was developed to gather detailed data on the following:

- Personal characteristics: sex, age, name of hospital, marital status, duration of career, and frequency of night shift per week.
- Medical history: personal and family history of chronic disease and non-communicable diseases including cancer, diabetes, hypertension, obesity, or any other chronic diseases, as well as use of medications and supplements and their types.
- Dietary habits and personal lifestyle: main meal, number of daily meals, skipped meals, and consumption of fast food.
- Dietary intake assessment: food frequency list method (Hammod, 2012) were used to estimate the dietary intake of foods (in g/day) expected to contain high levels of carcinogens (nitrosamine) commonly consumed by the medical staff such as processed meat (luncheon, corned beef, pastrami, sausage, hot dog, burger, and salami).The body weight of each participant was measured during the study to ensure accuracy.

#### Dietary intake of carcinogens

For dietary intake assessment, the study employed 24-hour dietary recalls for three successive days to collect accurate information on the average daily intake of each food item consumed by the participants, measured in grams per day (g/day). The dietary intake of carcinogens consumed by physicians, nurses, and pharmacists was estimated as follows: (1) average daily intake of each food item consumed for each subject of the study in g/day; (2) The results of food’s chemical analysis, which show the average amount of carcinogens in each gram of edible food; (3) An estimate of each subject’s carcinogens intake was compared to the tolerable daily intake established by The European Food Safety Authority (EFSA) for nitrates and nitrites^12^.

#### Part II: food samples

The rationale for choosing food items based on their commonality in food outlets is supported by market research, consumption patterns, and previous studies. Processed meats such as hot dogs, sausages, luncheon meats, corned beef, pastrami, burgers, and salami are among the most consumed fast food items, due to their convenience, taste, and affordability. Studies on dietary habits indicate that younger populations, especially those up to 39 years old, frequently consume these items, driven by busy lifestyles and a preference for quick, ready-to-eat options. An experimental study was conducted on the following food items: processed meat, luncheon, corned beef, pastrami, sausage, hot dog, burger, and salami. These samples are considered the most common fast-food items served in food outlets. Food samples were collected from various hypermarkets across Alexandria, ensuring a broad representation of brands and products by selecting hypermarkets in different locations known for high purchase power and a wide variety of processed meat products. Each product type was purchased in sufficient quantities to conduct multiple tests, ensuring reliability and reproducibility. Data collection involved meticulous documentation of each food sample, including the brand, manufacturing date, expiration date, and storage conditions. Upon purchase, the samples were transported to the laboratory under controlled temperature conditions to prevent any chemical alterations, stored at -20 °C until analysis, and then thawed at 4 °C for 24 h before testing. Each type of processed meat was homogenized to ensure uniformity, with a representative portion taken for analysis to ensure all parts of the product were equally represented. Nitrites were determined using the spectrophotometric method^13^.

#### Sample extraction

Ten grams of each product was ground well, placed in a glass beaker, heated in the water bath with distilled water, and shaken for a while, then cooled and filtered. The filtrate was collected and transferred into a test tube to identify nitrite and nitrate in samples. 1 mL of 0.5% sulfanilic acid and 1 mL of 2 mol/L hydrochloric acid solution were added and the solution was shaken thoroughly for 5 min to allow the diazotization reaction to complete. Then, 1 mL of 0.5% methyl anthranilate and 2 mL of 2 mol/L sodium hydroxide solution were added to form an azo dye, and the contents were diluted to 10 mL using water. After dilution to 10 mL with water, the absorbance of the red-colored dye was measured at 493 nm against the corresponding reagent blank and the calibration graph was constructed^14^.

#### Tools and instruments used

UV-VIS spectrophotometer (UNICO UV-Vis spectrophotometer (S12000)) Wp1001006081 with 1 cm quartz cell, analytical balance (Mettler Toledo), water bath (Griffin), filter paper, rubber ball, spatula, thermometer, mortar, and pestle and necessary glass-ware were used in this study. The visible spectrophotometry method used was based on the diazotation reaction of nitrite with aromatic primary amine compound coupled with N- (1-Naphthyl) and ethylene diamin dihydrochloride (NED). Nitrate is reduced to nitrite and then determined as nitrite^15^.

#### Dietary exposure calculation

The nitrosamine exposure was calculated by multiplying the nitrosamine concentration in the food by the corresponding daily intake of the food item (g/day). This was normalized by body weight (BW) to express exposure in µg/kg BW/day.

The equation used:

$$Exposure~\left( {\mu g/kg~BW/day} \right) = ~\frac{{\sum \left( {Nitrosa\min e~concentration~\left( {\mu g/g} \right) \times Food~\text{int} ake~\left( {g/day} \right)} \right)}}{{Body~weight~\left( {kg} \right)}}$$

The mean exposure across the population was then calculated to represent the typical exposure level.

#### Risk characterization using MOE

To characterize the risk of nitrosamine exposure, the Margin of Exposure (MOE) was calculated. MOE quantifies the level of concern associated with exposure to a genotoxic and carcinogenic compound. It is defined as the ratio of the benchmark dose lower confidence limit for a 10% response (BMDL10) to the estimated exposure:

$$~MOE = \frac{{BMDL10~\left( {\mu g/kg~BW/day} \right)}}{{Exposure~\left( {\mu g/kg~BW/day} \right))}}$$

#### Risk assessment of nitrosamine intake

The margin of exposure (MOE) value was calculated by comparing the mean value of nitrosamine (NA) exposure against the BMDL10 value of 29 µg kg − 1 BWday − 1^16^. The margin of exposure (MOE) was calculated by dividing BMDL10 value by mean exposure in µg kg − 1 BWday − 1 a MOE value ≥ 17,000 was derived for the exposure to NA known to be carcinogenic, indicating an exposure of low concern. For genotoxic compounds, EFSA has expressed that if the BMDL10 is 10,000 times higher than the exposure (i.e., MOE of 10,000), the exposure is of low concern^8^.

#### Ethical consideration

The experimental protocol was approved by the ethical committee of the High Institute of Public Health, Alexandria University. All methods were carried out following relevant guidelines and regulations. The research got the approval of the Ethics Committee of the High Institute of Public Health before conducting the research. The researchers comply with the International Guidelines for Research Ethics. A written consent was obtained from all study participants after an explanation of the purpose and benefits of the research. Anonymity and confidentiality were assured and maintained. There is no conflict of interest.

#### Statistical analysis of the data

Data were fed to the computer and analyzed using IBM SPSS software package version 20.0. **(**Armonk, NY: IBM Corp**)**, URL link: https://www.ibm.com/products/spss-statistics. Qualitative data were described using numbers and percentages. The Kolmogorov-Smirnov test was used to verify the normality of distribution Quantitative data were described using mean and standard deviation Significance of the obtained results was judged at the 5% level. The used tests were the Chi-square test for categorical variables, to compare between different groups, the Monte Carlo correction for chi-square when more than 20% of the cells have an expected count less than 5, and the F-test (ANOVA) for normally distributed quantitative variables, to compare between more than two groups, and Post Hoc test (Turkey) for pairwise comparisons.

## Results

Table 1 presents the personal characteristics of the studied groups. Participants aged 30 years or younger constituted the majority, accounting for 89.3%, 89.3%, and 88.6% of physicians, pharmacists, and nurses, respectively, with no significant difference among the groups. Males made up 39.8% (167 participants), while females comprised 60.2% (253 participants), again with no significant difference across the groups. Regarding marital status, most participants were single, representing 64.3% of the total, while 35.7% were married. There was a highly significant difference between groups in marital status. The majority of participants had been in their careers for five years or less, comprising 73.3% of the total, 75.7% of physicians, 74.3% of pharmacists, and 70% of nurses with no significant difference among the groups. Similarly, no significant difference was observed regarding the number of shifts worked. In terms of shift duration, most participants worked 12-hour shifts, with 84.5% overall 88.6% of physicians, 90% of pharmacists, and 75% of nurses reporting this schedule, and again, no significant difference among the groups was found. Table (1) also indicates that lunch was the main meal for the majority of participants, with 71.2% overall, 77.9, 67.9, and 67.9% among physicians, pharmacists, and nurses, respectively. The most commonly skipped meal was breakfast. It was noted that all participants consumed more than one meal per day, with the majority having three meals daily (77.1, 73.6, and 79.3%) among physicians, pharmacists, and nurses, respectively.

**Table 1:** Personal characteristics and dietary habits of study participants.

I. Personal characteristics:	Total (*n* = 420)		Profession						χ^2^	*p*
			Physician (*n* = 140)		Pharmacist (*n* = 140)		Nurse (*n* = 140)			
	No.	%	No.	%	No.	%	No.	%		
Age										
< 30	374	89.0	125	89.3	125	89.3	124	88.6	0.049	0.976
30–39	46	11.0	15	10.7	15	10.7	16	11.4		
Sex										
Male	167	39.8	66	47.1	54	38.6	47	33.6	5.507	0.064
Female	253	60.2	74	52.9	86	61.4	93	66.4		
Marital status										
Single	270	64.3	107	76.4	99	70.7	64	45.7	32.542^*^	< 0.001^*^
Married	150	35.7	33	23.6	41	29.3	76	54.3		
Divorced	0	0.0	0	0.0	0	0.0	0	0.0		
Widowed	0	0.0	0	0.0	0	0.0	0	0.0		
Duration of career										
< 5	308	73.3	106	75.7	104	74.3	98	70.0	1.742	0.783
5 ≥ 10	94	22.4	29	20.7	31	22.1	34	24.3		
10+	18	4.3	5	3.6	5	3.6	8	5.7		
Number of shifts per week										
1	63	15.0	19	13.6	23	16.4	21	15.0	6.385	0.604
2	46	11.0	14	10.0	12	8.6	20	14.3		
3	106	25.2	36	25.7	33	23.6	37	26.4		
4	118	28.1	39	27.9	38	27.1	41	29.3		
+ 5	87	20.7	32	22.9	34	24.3	21	15.0		
Duration of each shift (hours)										
6	24	5.7	0	0.0	12	8.6	12	8.6	31.002^*^	< 0.001^*^
12	355	84.5	124	88.6	126	90.0	105	75.0		
24	41	9.8	16	11.4	2	1.4	23	16.4		
Main meal										
Lunch	299	71.2	109	77.9	95	67.9	95	67.9	4.551	0.103
Dinner	121	28.8	31	22.1	45	32.1	45	32.1		
Number of daily meals/snacks										
Two	36	8.6	10	7.1	15	10.7	11	7.9	3.525	0.741
Three	322	76.7	108	77.1	103	73.6	111	79.3		
Four	37	8.8	12	8.6	12	8.6	13	9.3		
Five	25	6.0	10	7.1	10	7.1	5	3.6		

χ^2^: Chi square test.

*: Statistically significant at *p* ≤ 0.05.

Table 2 shows the medical history of the studied groups. It was noticed that most cases had no presence of chronic diseases and represented 87.1, 89.3, 84.3, and 87.9% of the total, physicians, pharmacists, and nurses respectively. The majority of participants had no presence of allergies and all the chronic illnesses were among first-degree relatives of all participants. Participants who used multivitamins and minerals represented 68.6, 77.1, 61.4 and 67.1% of the total, physicians, pharmacists, and nurses.

**Table 2:** Medical history of study participants.

II. Medical history (Personal history)	Total (*n* = 420)		Profession						χ^2^	*p*
			Physician (*n* = 140)		Pharmacist (*n* = 140)		Nurse (*n* = 140)			
	No.	%	No.	%	No.	%	No.	%		
Presence of chronic Diseases										
No	366	87.1	125	89.3	118	84.3	123	87.9	2.498	^MC^p 0.887
Diabetes	23	5.5	6	4.3	10	7.1	7	5.0		
Hypertension	18	4.3	5	3.6	8	5.7	5	3.6		
Obesity	13	3.1	4	2.9	4	2.9	5	3.6		
Presence of allergies										
No	368	87.6	132	94.3	120	85.7	116	82.9	9.130^*^	0.010^*^
Yes	52	12.4	8	5.7	20	14.3	24	17.1		
Use of any medications										
Don’t use any drugs	340	81.0	121	86.4	104	74.3	115	82.1	7.214	0.302
Anti-diabetic drugs	23	5.5	6	4.3	10	7.1	7	5.0		
Antihypertensive	15	3.6	4	2.9	7	5.0	4	2.9		
Other	42	10.0	9	6.4	19	13.6	14	10.0		
Use of any supplements										
Don’t use any supplements	328	78.1	115	82.1	105	75.0	108	77.1	2.607	0.626
Vitamins	58	13.8	17	12.1	22	15.7	19	13.6		
Multivitamins and minerals	34	8.1	8	5.7	13	9.3	13	9.3		

χ^2^ Chi-square test, *MC *Monte Carlo.

*: Statistically significant at *p* ≤ 0.05.

Table 3 shows the Comparison among the studied groups according to the total score of knowledge about carcinogens. It was noticed that 46.9% of total participants showed poor knowledge and 45.7% had fair knowledge about carcinogens. Participants who had good Knowledge about carcinogens were 7.4% of the total participants. Physicians scored a good level of knowledge about carcinogens in 29 participants (20.7%) while pharmacists’ good knowledge was evaluated in only two participants (1.4%).

**Table 3:** Total score of knowledge about carcinogens among studied groups.

Total score of Knowledge about carcinogens:	Total (*n* = 420)		Profession						*P*
			Physician (*n* = 140)		Pharmacist (*n* = 140)		Nurse (*n* = 140)		
	No.	%	No.	%	No.	%	No.	%	
Poor knowledge(< 50%)	197	46.9	3	2.1	75	53.6	119	85.0	< 0.001^*^
Fair knowledge(50 − 75%)	192	45.7	108	77.1	63	45.0	21	15.0	
Good knowledge(> 75% )	31	7.4	29	20.7	2	1.4	0	0.0	
Mean ± SD.	13.08 ± 3.83		17.01 ± 2.23		12.31 ± 2.52		9.91 ± 2.55		< 0.001^*^

χ^2^: Chi-square test F: F for One way ANOVA test.

Table 4 shows a Comparison between the studied groups according to dietary intake assessment. It was noticed that there was a significant difference among the three studied groups according to the dietary intake assessment (*p* < 0.05) except for Breakfast cereals intake (*p* = 0.295), Fried Chicken (*p* = 0.773), Biscuits (*p* = 0.057), and pate (*p* = 0.075).

**Table 4:** Comparison between the studied groups according to dietary intake assessment.

V. Dietary intake assessment	Total (*n* = 420)		Profession						χ^2^	*P*
			Physician (*n* = 140)		Pharmacist (*n* = 140)		Nurse (*n* = 140)			
	No.	%	No.	%	No.	%	No.	%		
Hydrogenated fats										
Not consumed	42	10.0	29	20.7	6	4.3	7	5.0	82.486^*^	< 0.001^*^
Weekly	33	7.9	20	14.3	4	2.9	9	6.4		
3–4 times per week	189	45.0	24	17.1	91	65.0	74	52.9		
Daily	156	37.1	67	47.9	39	27.9	50	35.7		
Butter										
Not consumed	85	20.2	32	22.9	26	18.6	27	19.3	28.431^*^	< 0.001^*^
Weekly	269	64.0	99	70.7	74	52.9	96	68.6		
3–4 times per week	66	15.7	9	6.4	40	28.6	17	12.1		
Daily	0	0.0	0	0.0	0	0.0	0	0.0		
Luncheon										
Not consumed	36	8.6	21	15.0	13	9.3	2	1.4	62.756^*^	< 0.001^*^
Weekly	18	4.3	0	0.0	16	11.4	2	1.4		
3–4 times per week	187	44.5	68	48.6	37	26.4	82	58.6		
Daily	179	42.6	51	36.4	74	52.9	54	38.6		
Corned beef										
Not consumed	78	18.6	31	22.1	20	14.3	27	19.3	15.741^*^	0.015^*^
Weekly	42	10.0	12	8.6	12	8.6	18	12.9		
3–4 times per week	222	52.9	63	45.0	91	65.0	68	48.6		
Daily	78	18.6	34	24.3	17	12.1	27	19.3		
Pastrami										
Not consumed	80	19.0	35	25.0	23	16.4	22	15.7	61.870^*^	< 0.001^*^
Weekly	51	12.1	12	8.6	22	15.7	17	12.1		
3–4 times per week	152	36.2	25	17.9	77	55.0	50	35.7		
Daily	137	32.6	68	48.6	18	12.9	51	36.4		
Sausage										
Not consumed	91	21.7	39	27.9	20	14.3	32	22.9	38.590	< 0.001^*^
Weekly	171	40.7	32	22.9	72	51.4	67	47.9		
3–4 times per week	136	32.4	56	40.0	47	33.6	33	23.6		
Daily	22	5.2	13	9.3	1	0.7	8	5.7		
Hot dog										
Not consumed	92	21.9	45	32.1	15	10.7	32	22.9	77.799	< 0.001^*^
Weekly	147	35.0	63	45.0	33	23.6	51	36.4		
3–4 times per week	163	38.8	21	15.0	91	65.0	51	36.4		
Daily	18	4.3	11	7.9	1	0.7	6	4.3		
Burger										
Not consumed	97	23.1	43	30.7	16	11.4	38	27.1	39.844^*^	< 0.001^*^
Weekly	82	19.5	20	14.3	39	27.9	23	16.4		
3–4 times per week	212	50.5	59	42.1	84	60.0	69	49.3		
Daily	29	6.9	18	12.9	1	0.7	10	7.1		
Salami										
Not consumed	158	37.6	70	50.0	31	22.1	57	40.7	43.903^*^	< 0.001^*^
Weekly	225	53.6	70	50.0	83	59.3	72	51.4		
3–4 times per week	37	8.8	0	0.0	26	18.6	11	7.9		
Daily	0	0.0	0	0.0	0	0.0	0	0.0		

χ^2^: Chi-square test* MC* Monte Carlo *: Statistically significant at *p* ≤ 0.05.

Table 5 demonstrated that hotdogs had the highest nitrosamine (159.24 ± 87.99 µg/g) followed by pastrami then Salami (33.81 ± 60.41 and 32.02 ± 8.15 µg/g) respectively, while luncheon had the lowest content (18.53 ± 11.80 µg/g). Hotdog had the highest consumption pattern followed by Corned beef then Sausage 0.02126, 0.00638, and 0.00516, while luncheon had the lowest concentration and pattern 18.53 ± 11.80 µg/g, and 0.00141 µgkg-1BW sd-1 respectively.

**Table 5:** Concentration of nitrosamine (µg/g) in different processed meat available in the Alexandria market.

Food items	Nitrosamine level in µg /g					
	No. of products	Mean ± SD.	Min. – Max.	Median	IQR	Dietary Intake among medical staff (µgkg-1BW sd-1)
Luncheon	11	18.53 ± 11.80	9.57–46.42	12.69	10.97–21.88	0.00141
Corned Beef	7	30.16 ± 7.20	22.70–44.04	29.17	25.27–32.34	0.00638
Pastrami	9	33.81 ± 60.41	7.47–194.17	10.32	9.86–22.0	0.00410
Sausage	10	27.36 ± 46.65	2.36–156.91	12.86	7.74–15.09	0.00516
Hot dog	9	159.24 ± 87.99	54.40–360.16	148.0	111.0–181.6	0.02126
Burger	10	27.14 ± 20.71	2.37–74.81	24.23	14.77–38.12	0.00426
Salami	5	32.02 ± 8.15	23.64–44.12	31.13	25.89–35.31	0.00269

Tables 6 and 7 shows the total dietary intake of Nitrosamine among medical staff during their night shift, it was noticed that pharmacists had the highest intake (0.0507 ± 0.0116) followed by nurses (0.0442 ± 0.0158), while physicians had the lowest intake 0.0408 ± 0.0156 (µg/kgBW/d). There was a statistically significant difference among the studied groups according to dietary intake of Nitrosamine among medical staff (*p* < 0.001). The table also showed that physicians are the most group at risk from the consumption of Nitrosamine based on MOE calculations with MOE 710. All groups and total samples showed no public health concern from dietary intake of Nitrosamine based on MOE calculations which were all below 10000.

**Table 6:** Comparison between the different studied groups according to total dietary intake and margin of exposure (µg/kg) of nitrosamine among medical staff.

T. Nitrosamine	Total (*n* = 420)	Profession			F	*P*
		Physician (*n* = 140)	Pharmacist (*n* = 140)	Nurse (*n* = 140)		
Dietary intake of nitrosamine among medical staff (µgkg-1BW sd-1)	0.0453 ± 0.015	0.0408 ± 0.0156	0.0507 ± 0.0116	0.0442 ± 0.0158	16.843^*^	< 0.001^*^
Pairwise	p_1_ < 0.001^*^,p_2_ = 0.125,p_3_ = 0.001^*^					

*SD* Standard deviation,* F* F for One way ANOVA test, Pairwise comparison bet. each 2 groups was done using a Post Hoc Test (Tukey).

p: p-value for comparing between the studied groups.

p_1_: p-value for comparing between physician and pharmacist.

p_2_: p-value for comparing between physician and nurse.

p_3_: p-value for comparing between pharmacist and nurse.

*: Statistically significant at *p* ≤ 0.05.

**Table 7:** Comparison between the different studied groups according margin of exposure (µg/kg) of nitrosamine among medical staff.

T. Nitrosamine	Total (*n* = 420)	Profession		
		Physician (*n* = 140)	Pharmacist (*n* = 140)	Nurse (*n* = 140)
MOE BMDL10 = 29 µgkg^**− 1**^BWday^**− 1**^	640^**^	710^**^	571^**^	656^**^
P90-MOE	0.062–468**	0.0558- 492**	0.064089- 452**	0.06235- 465**
P95-MOE	0.0659–440**	0.06208- 467**	0.06896- 420**	0.066859- 434**

^**^: For carcinogenic and genotoxic effects MOE below 10,000 would be of no public health concern.

*BMDL* Benchmark dose level.

## Discussion

The data in Table 1 aligns with Arafa, and Eshak (17) on night and rotating shift work and its correlation with cancer risk among Japanese men and women. Their research showed that 44.86% of participants were men and 55.14% were women, indicating women might be more affected by carcinogen exposure^17^. An early analysis utilizing data from the JACC Study showed that for women, the age-adjusted correlation between daytime sleep (yes vs. no) and death from liver cancer was substantial. In contrast, men were more likely to have a significant HR (95% CI) of 1.23 (1.17, 2.24) than women (0.97, 1.55)^18^. Concerning the duration of their career and the number of shifts they worked did not differ significantly among groups as the majority of participants worked for five years or less, these findings are consistent with the study conducted by Arafa, and Eshak (17), which reported that most males worked during the day, while women were more likely to work during the day as well, but a smaller percentage worked at night or rotating shifts^19^. Another study^20^ found that older adults who took daytime naps had a higher risk of developing liver cancer, while younger adults took brief naps during the day to get ready for work. It has been shown that short daytime naps have no harmful effects, while longer daytime naps can disrupt sleep cycles and increase the risk of cardiovascular disease and cancer. This disturbance is linked to a malfunctioning circadian clock^21^.

Most participants had three meals a day, supported by sensitivity analysis showing that the link between daytime napping and liver cancer risk weakened after excluding housewives or unemployed individuals^22^.Protein consumption was most effective for increasing calorie intake late in the afternoon on workdays, while fat intake was highest in the early morning and after midday on rest days. Carbohydrates were most significant for energy intake early in the morning or late at night^23^.Night shift workers often ate fewer full meals, relying on fast food and grazing during shifts, and consuming smaller meals at longer intervals^24,25^.

It is recommended that adults should consume 4–5 meals every day according to the Polish nutritional guidelines^26^. Eating smaller meals frequently helps prevent hypoglycemia, which can occur after consuming a large meal while at work and results in hyperinsulinemia^27^. A second survey showed that more than 60% of healthcare professionals reported eating at least 4 meals per day. The number of meals did not significantly change based on the work mode of the respondents, and only 8% of the respondents reported consuming 1–2 meals per day^28^.

Polish guidelines recommend 4–5 meals daily to prevent hypoglycemia, which can result from eating large meals at work and lead to hyperinsulinemia. More than 60% of healthcare professionals reported eating at least four meals per day, with no significant change based on work mode. Sińska et al.^29^ found that 50% of nurses ate 4–5 meals per day, while Bielak’s research noted that 25% of nurses skipped breakfast and 30% had only 1–2 meals daily^30^.Skipping meals can cause low blood glucose levels, light-headedness, fatigue, and slower muscle recovery. Also, prolonged breaks between meals cause the body to produce excess ghrelin, leading to overeating.

### Nitrites content and dietary intake of nitrosamine

The presence of nitrate, nitrite, and nitrosamines in food items necessitates a comprehensive assessment of the potential health hazards associated with their daily dietary consumption^31,32^. Assessing nitrate, nitrite, and nitrosamine levels in food is crucial for understanding potential health risks. This study evaluated nitrosamine intake among medical professionals during night shifts and found significant differences in consumption across groups (*p* = 0.001). Moazeni, Heidari ^33^recommended daily intakes of 4.22 mg/kg/day nitrate and 0.09 mg/kg/day nitrite from food, with additional intake from drinking water. Ghaffari, Nasseri ^31^reported average daily nitrate intake from fruits and vegetables in Iran as 1.94 ± 0.95 mg/kg/day in high-risk areas and 1.98 ± 1.06 mg/kg/day in low-risk areas for stomach cancer.

In the current study, medical professionals were evaluated for their total Nitrosamine intake during night shifts. More importantly, the study demonstrated a statistically significant difference in Nitrosamine consumption among medical professionals across the different groups (*p* = 0.001). A study recommends daily intakes of 4.22 mg/kg/day nitrate 0.09 mg/kg/day nitrite from food for three days, and 0.43 mg/kg/day nitrate and 0.005 mg/kg/day nitrite from drinking water stated the average recommended daily intake of nitrate from fruits and vegetables in Iran to be 1.94 ± 0.95 mg/kg/day in high-risk locations and 1.98 ± 1.06 mg/kg/day in low-risk locations for stomach cancer.

Table 5 shows nitrite levels in food products from various countries. All samples were below the (EC) No. 601/2014 limits, except Estonian sausage, which exceeded the limit. France and Russia had the lowest nitrite content, highlighting the need for ongoing monitoring and regulation. The WHO sets a maximum nitrite level of 10 µg/kg for processed meats. Factors like nitrite content, temperature, and storage affect nitrosamine formation. This study found that NMTCA levels in all samples exceeded the WHO limit, with hotdogs showing the highest nitrosamine content (159.24 ± 87.99 µg/g), followed by pastrami and salami. Luncheon had the lowest nitrosamine content (18.53 ± 11.80 µg/g)^16,34–37^.

Regarding the minimum content of nitrosamine, it was found in Sausage at 2.36 µg/g and Burgers at 2.37 µg/g. Based on previous research conducted by Herrmann, and Duedahl-Olesen (16), the amounts of nitrosamines obtained were likewise variable. NTCA has the greatest nitrosamine concentration (4030 g/kg). The findings of this investigation are consistent with prior studies in which NDEA levels were not discovered in all tested samples. However, nitrosamine levels in this research are greater than in earlier investigations. NTCA has the highest nitrosamine content at 4227.492 g/kg (smoked beef Chiefs). Smoked beef products had the greatest amounts of NMTCA and NTCA. This is also consistent with Herrmann, Duedahl-Olesen’s (16) earlier research where the levels of nitrosamines reach the maximum level of 2034–4030 µg/kg.

During the smoking process, smoked beef samples are exposed to high temperatures which results in the production of two nitrosamines. The nitrite content, temperature, storage conditions, and the presence of catalysts or inhibitors can also contribute to their formation. The quantity of nitrite and amine molecules present determines the ease of nitrosamine formation. While NMTCA and NTCA (non-volatile nitrosamine) compounds have not exhibited toxicological characteristics, non-volatile nitrosamines are considered to be mild carcinogens. However, there is insufficient evidence to assess their toxicological effects. To reduce cancer risk, the levels of NMTCA and NTCA nitrosamines in processed meat products must be monitored^16,38^.

Regarding MOE of nitrosamine, the recent study agreed with a study conducted in Denmark to assess the risk of consuming nitrosamine-containing products. The margin of exposure calculated based on BMDL10 of 29 µgkg − 1 BWday^− 1^ was found to be of no public health concern (below 10000), which agreed with the MOE calculated in our study using the same BMDL10 value^38,39^. EFSA Panel on Contaminants in the Food Chain calculated the MOE for Nitrosamine consumption using a different BMDL10. The lower confidence limit of the benchmark dose at 10% (BMDL10) was 10 µg/kg body weight (bw) per day, derived from the incidence of rat liver tumors (benign and malignant) used for the calculation of Margin of exposure. The calculated MOE ranged from 48 to 3337 for p 95 which was far from the MOE calculated in the present study due to the use of different BMDL10 and different age categories of the sample used but agreed on both MOE are of low public health concern (below 10000)^40^. Table (8) will summarize the results of various studies that quantified nitrites in different processed meat products worldwide.

**Table 8:** Comparative analysis of nitrite levels in processed meats across different countries obtained from various literature sources and comparison with the Present study.

Country	Year	Number of samples	Nitrite (ppm)	Sample type	Results of the present study mg kg-1(ppm) (Min-Max)	Nitrites legal limits reported in Regulation (EC) No. 601/2014.
Finland^41^	1994		48 mg kg-1	Sausages		
France^42^	1995, 2002	3,112	0 to 9 mg kg-1	Raw, dried, cooked, and cured meat products	Luncheon (9.57–46.42) Corned Beef (22.70–44.04) Pastrami (7.47–194.17) Sausage (2.36–156.91) Hot dog (54.40–360.16) Burger (2.37–74.81) Salami (23.64–44.12)	Heat and non-heat-treated processed meat (150 ppm) Traditionally cured meat products with specific provisions concerning nitrites and nitrates (50–180)
Denmark^43^	1995–2006		60 mg/kg	Most products		
United Kingdom^44^	1997	200	0.2–123 mg/kg 0.2–170 mg/kg	Bacon and other meat products		
Russia^45^	1999	186	0.2–9.1 mg/kg	Meat products		
Greece^46^	2000	30	0.85-189.65 mg/kg	Pastrami		
Estonia^47^	2000–2004	189	100 and 250 mg/kg	Sausage		
Ireland^48^	2001, 2002	147	0–20 mg kg-1 (36%) 20–29 mg/kg (20%) 30–39 mg/kg (12%) 40–49 mg/kg (7%)	Bacon		
Germany,^48^	2001, 2002	116	below 20 mg/kg	Cured meat		
Belgium^49^	2002	75	below 20 mg/kg	Cured meat		
five cities of USA^50^	2009		7 ppm	Cured meat products (hot dogs, bacon and hams		

## Conclusions and recommendations

The current experimental study provides an overview of the nitrosamine content found in processed meat products that are commonly consumed by the general public and pose major public health concerns. The study results showed that there was a statistically significant difference in nitrosamine intake between the studied groups, based on their dietary habits, among medical staff. The study recommends establishing good eating routines for night-shift employees, as their eating habits may harm their health. To reduce nitrosamine intake in processed foods, the food industry should gradually reformulate its products while maintaining their organoleptic properties to prevent consumer rejection. Finally, the study suggests that restaurants should gradually reduce the amount of nitrosamine in their meals.

### Study limitations

The study offers valuable insights into nitrosamine exposure among physicians, pharmacists, and nurses, though it has some limitations. Focusing on these specific healthcare professionals may limit the applicability of the findings to other occupational groups or the general population. The reliance on self-reported dietary data introduces potential biases, such as recall errors and reporting inaccuracies, which could affect result accuracy. Additionally, the cross-sectional design captures data at only one point in time, potentially missing variations in long-term exposure or health outcomes. Despite these limitations, the study significantly enhances our understanding of carcinogen exposure, particularly for healthcare workers during night shifts.

## Electronic supplementary material

Below is the link to the electronic supplementary material.

Supplementary Material 1

## Data Availability

The data used to support the findings of this study can be made available by the corresponding author upon request.
