# Supplementary material for: Dietary intake and risk assessment of nitrosamine in processed meat products among medical staff during their night shift
Source: Sci Rep. 2025 Jan 14;15:1898. doi: 10.1038/s41598-024-84059-y (PMC11730967; doi:10.1038/s41598-024-84059-y)
Supplement: Supplementary file 1 — Supplementary Material 1 [file 41598_2024_84059_MOESM1_ESM.doc]

**
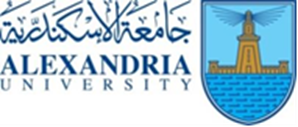
**

**High Institute of Public Health**

**Nutrition Department**

**Serial number:**

**Date: / /**

**Questionnaire for Alexandria University Hospitals medical staff to assess dietary intake of Some Carcinogens during their night shift**

Please answer the following questions, putting in mind that your answers should reflect your **current practices**

**Name: ………………………………………………………..**

**Telephone Number:………………………………………....**

**I. Personal characteristics:**

|  |
| --- |

**1. Age:**

|  |
| --- |

**2. Sex:** Male (1) Female (2)

|  |
| --- |

**3. Name of hospital: ………………**

**4. Marital Status:** Single (1) Married (2) Divorced (3) Widowed (4)

|  |
| --- |

**5. Number of Children: ……..**

|  |
| --- |

**6. Family Size: ……….**

|  |
| --- |

**7. Place of Residence**: Urban (1) Rural (2)

|  |
| --- |

**8. Profession :** physician (1) pharmacist (2) nurse (3)

|  |
| --- |

**9. Date of Start of Residency (D/M/Y):**

|  |
| --- |

**10. Duration of career:………………..**

|  |
| --- |

**11. Number of shifts per week :.....................**

**12. Duration of each shift (hours).......................**

|  |
| --- |

|  |
| --- |

**II. Medical history:**

**Personal history**

**13. Presence of chronic Diseases:**

Cancer (1) Diabetes (2) Hypertension (3) Obesity (4) Other…….(5) No (0)

**14. Presence of allergies:**

Yes (1) No (0)

**15. Use of any medications:**

Chemotherapy (1) Anti-diabetic drugs (2) Antihypertensive (3) other…(4)

Don’t use any drugs (-1)

**16. Use of any supplements :**

Vitamins (1) Minerals (2) Multivitamins and minerals (3)

Don’t use any supplements (-1)

**Family history:**

**17. Degree of relativity:**

First degree (mother or father) (1) Second degree (2) Third degree (3) none (-1)

**18.** **Presence of chronic diseases in family history:**

Cancer (1) Diabetes (2) Hypertension (3) Obesity (4) Other…….(5)
No (0)

**19. Presence of allergies in family history:**

Yes (1) No (0)

**20. Use of any medications:**

Chemotherapy (1) Anti-diabetic drugs (2) Antihypertensive (3) Other…(4)

Don’t use any drugs (-1)

**21. Use of any supplements:**

Vitamins (1) Minerals (2) Multivitamins and minerals (3)

Other…(4) Don’t use any supplements (-1)

**III. Dietary habits and personal lifestyle:**

**22. Main meal:**

Breakfast (1) Lunch (2) Dinner (3)

**23. Numbers of daily meals / snacks**:

One (1) Two (2) Three (3) Four (4) Five (5)

**24. Skipped meal:** Breakfast (1)

Lunch (2) Dinner (3) Snack (4) None (0)

**25. Type of cooking fats:** hydrogenated fat

(سمن صناعي) (1) Butter (الزبدة الصفراء) (2) Ghee (السمن البلدي ( (3)Vegetable oils

(زيت صويا,(زيت زيتون, زيت عبادالشمس,زيت جوز الهند Other (4)……….. Don’t know(5)

**26.Amount of fats per day:**

One teaspoon (1) One tablespoon (2) Two tablespoon (3) More than two (4)

Don’t know (5)

**27. Type of food you always eating outside of home during your night shifts:**

Fast food (1) Salted food (3) Artificial sweetener (4)

Processed food (5) Homemade food (6)

**28.Ordering fast-food (from restaurants) per week:**

Once (1) Twice (2) Three times or more (3) Daily (4)

None (0)

**29.Type of smoking:**

Cigarettes (1) Shisha (2) Passive smoker (3) I am not smoker (0)

**30.Smoking per day:**

Once (1) Twice (2) Three times or more (4) Non _smoker (-1)

**31.Type of physical activity per week:**

Every day (1) More than three times (2) Three times (3) None (0)

**32. Type of physical activity:**

I don’t really exercise now (-1) Walking (1) Others (please specify) (2) ……………

**IV. Knowledge about carcinogens:**

| 33. A carcinogen is an agent with the capacity to cause cancer in humans. | True (1) | False (0) | Don’t know (0) |  |
| --- | --- | --- | --- | --- |
| 34. Acrylamide isn’t type of carcinogens. | True (0) | False (1) | Don’t know  (0) |  |
| 35. Trans fats is type of carcinogens. | True (1) | False (0) | Don’t know (0) |  |
| 36. Nitrosamine may cause cancer because it is type of carcinogen | True (1) | False (0) | Don’t know (0) |  |
| 37. Chips and French fries are most common source of acrylamide and trans fat. | True (1) | False (0) | Don’t know (0) |  |
| 38. Transfat and acrylamide are present in fine bakery | True (1) | False (0) | Don’t know (0) |  |
| 39. Rice and macaroni may contain acrylamide. | True (1) | False (0) | Don’t know (0) |  |
| 40. Nitrosamine is present in processed meat . | True (1) | False (0) | Don’t know (0) |  |
| 41. luncheon and pastrami aren’t healthy to eat every day. | True (1) | False (0) | Don’t know |  |
| 42. Paton sale’, croissant , pate` aren’t healthy snacks its contains trans fat and acrylamide . | True (1) | False (0) | Don’t know (0) |  |
| 43. You should reduce biscuits ,chocolate, crisps for healthy life because these snacks may contain some carcinogens like acrylamide and trans fat. | True (1) | False (0) | Don’t know (0) |  |
| 44. Eating whole grain bread is more healthy than white bread and toast . | True (1) | False (0) | Don’t know (0) |  |
| 45. Popcorn is totally healthy snack it doesn’t have any carcinogen. | True (0) | False (1) | Don’t know |  |
| 46. Home made meals are more safe. | True (1) | False (0) | Don’t know (0) |  |
| 47. Cereals are healthier breakfast and don’t have any carcinogens. | True (0) | False (1) | Don’t know |  |
| 48. You should eat vegetables and fruits every day. | True (1) | False (0) | Don’t know (0) |  |
| 49. Exercising is very important for healthy life. | True (1) | False (0) | Don’t know (0) |  |
| 50. Smoking may cause cancer and may cause death. | True (1) | False (0) | Don’t know (0) |  |
| 51. Eating raw nuts is more healthy than roasted nuts because roasted nuts may contain acrylamide. | True (1) | False (0) | Don’t know (0) |  |
| 52. Milk chocolate may contain acrylamide. | True (1) | False (0) | Don’t know (0) |  |
| 53. Dark chocolate 80% is healthier than milk chocolate . | True (1) | False (0) | Don’t know (0) |  |
| 54. Dark chocolate 80% is a very good antioxidant. | True (1) | False (0) | Don’t know (0) |  |
| 55. Falafel and fried eggplants are containing trans fat. | True (1) | False (0) | Don’t know (0) |  |
| 56. You should eat fruits and vegetables especially with high antioxidants. | True (1) | False (0) | Don’t know (0) |  |
| 57. Blueberries, strawberries , red cabbage and pecans they are the top foods with high antioxidants. | True (1) | False (0) | Don’t know (0) |  |

**V. Dietary intake assessment:**

**Consumption of the following types of foods and their amounts per week during your night shift:**

|  | ***Not consumd(0)*** | **Weekly(1)** | **3-4 times per week (2)** | **Daily (3)** | **Household** | **G|d** | **Code** |
| --- | --- | --- | --- | --- | --- | --- | --- |
| 58.Chip (شبيسي) |  |  |  |  |  |  |  |
| 59.French fries |  |  |  |  |  |  |  |
| 60.Falafel |  |  |  |  |  |  |  |
| 61.Fried eggplant |  |  |  |  |  |  |  |
| 62.Crisps(كونو,ذرة  محمص,شيتوس) |  |  |  |  |  |  |  |
| 63.Popcorn |  |  |  |  |  |  |  |
| 64.Breakfast cereals |  |  |  |  |  |  |  |
| 65.Fried chicken |  |  |  |  |  |  |  |
| 66.White bread |  |  |  |  |  |  |  |
| 67.Whole grain |  |  |  |  |  |  |  |
| 68.Rice |  |  |  |  |  |  |  |
| 69.Macaroni |  |  |  |  |  |  |  |
| 70.Toast |  |  |  |  |  |  |  |
| 71.Baton sale` |  |  |  |  |  |  |  |
| 72.Cookies |  |  |  |  |  |  |  |
| 73.Cakes |  |  |  |  |  |  |  |
| 74.Biscuits |  |  |  |  |  |  |  |
| 75.Pate` |  |  |  |  |  |  |  |
| 76.Croissant |  |  |  |  |  |  |  |
| 77.Shawerma |  |  |  |  |  |  |  |
| 78.Liver |  |  |  |  |  |  |  |
| 79.Pizza |  |  |  |  |  |  |  |
| 80.Roasted nuts |  |  |  |  |  |  |  |
| 81.Chocolates |  |  |  |  |  |  |  |
| 82.Hydrogenated fats |  |  |  |  |  |  |  |
| 83.Butter |  |  |  |  |  |  |  |
| 84.Lunchon |  |  |  |  |  |  |  |
| 85.Corned beef |  |  |  |  |  |  |  |
| 86.Pastarmi |  |  |  |  |  |  |  |
| 87.Sausge |  |  |  |  |  |  |  |
| 88.Hot dog |  |  |  |  |  |  |  |
| 89.Burger |  |  |  |  |  |  |  |
| 90.Salami |  |  |  |  |  |  |  |

| 91. Weight in kg |  |  |  |  |  |  |  |
| --- | --- | --- | --- | --- | --- | --- | --- |
